# Circadian Regulation Does Not Optimize Stomatal Behaviour

**DOI:** 10.3390/plants9091091

**Published:** 2020-08-25

**Authors:** Víctor Resco de Dios, William R.L. Anderegg, Ximeng Li, David T. Tissue, Michael Bahn, Damien Landais, Alexandru Milcu, Yinan Yao, Rachael H. Nolan, Jacques Roy, Arthur Gessler

**Affiliations:** 1 School of Life Science and Engineering, Southwest University of Science and Technology, Mianyang 621010, China; yinanyao@swust.edu.cn; 2 Department of Crop and Forest Sciences-AGROTECNIO Center, University of Lleida, 25198 Lleida, Spain; 3 School of Biological Sciences, University of Utah, Salt Lake City, UT 84112, USA; anderegg@utah.edu; 4 Hawkesbury Institute for the Environment, Western Sydney University, Penrith, NSW 2751, Australia; Ximeng.Li@westernsydney.edu.au (X.L.); D.Tissue@westernsydney.edu.au (D.T.T.); Rachael.Nolan@westernsydney.edu.au (R.H.N.); 5 Department of Ecology, University of Innsbruck, 6020 Innsbruck, Austria; Michael.Bahn@uibk.ac.at; 6 Ecotron Européen de Montpellier, CNRS, 34980 Montferrier-sur-Lez, France; Damien.LANDAIS@cnrs.fr (D.L.); alexandru.milcu@cnrs.fr (A.M.); jacques.roy@cnrs.fr (J.R.); 7 Centre d’Ecologie Fonctionnelle et Evolutive (CEFE), CNRS, UMR 5175, Université de Montpellier, Université Paul Valéry, EPHE, IRD, 34293 Montpellier, France; 8 Forest Dynamics, Swiss Federal Institute for Forest, Snow and Landscape Research WSL, 8903 Birmensdorf, Switzerland; arthur.gessler@wsl.ch; 9 Institute of Terrestrial Ecosystems, ETH Zurich, 8092 Zurich, Switzerland

**Keywords:** adaptations, bean, cotton, ecological strategies, gas exchange, leaf

## Abstract

The circadian clock is a molecular timer of metabolism that affects the diurnal pattern of stomatal conductance (*g*_s_), amongst other processes, in a broad array of plant species. The function of circadian *g*_s_ regulation remains unknown and here, we test whether circadian regulation helps to optimize diurnal variations in stomatal conductance. We subjected bean (*Phaseolus vulgaris*) and cotton (*Gossypium hirsutum*) canopies to fixed, continuous environmental conditions of photosynthetically active radiation, temperature, and vapour pressure deficit (free-running conditions) over 48 h. We modelled *g*_s_ variations in free-running conditions to test for two possible optimizations of stomatal behaviour under circadian regulation: (i) that stomata operate to maintain constant marginal water use efficiency; or (ii) that stomata maximize C net gain minus the costs or risks of hydraulic damage. We observed that both optimization models predicted *g*_s_ poorly under free-running conditions, indicating that circadian regulation does not directly lead to stomatal optimization. We also demonstrate that failure to account for circadian variation in *g*_s_ could potentially lead to biased parameter estimates during calibrations of stomatal models. More broadly, our results add to the emerging field of plant circadian ecology, where circadian controls may partially explain leaf-level patterns observed in the field.

## 1. Introduction

Circadian rhythms regulate the temporal pattern of the transcription of ~30% of the plant genome [1]. Diurnal variation in photosynthesis and stomatal conductance, among other processes such as growth [2] and respiration [3], is affected by circadian regulation. Current estimates indicate that, averaged across species, circadian regulation drives 15–25% of the daytime oscillation in carbon assimilation (*A*) [4], at least under some environmental conditions. Resonance between circadian rhythms in gas exchange and environmental cues has been documented to increase plant growth [5,6,7,8,9] because circadian regulation underlies the temporal partitioning and synchronization of different processes associated with carbon metabolism. Relationships between the circadian clock and photosynthesis are bidirectional such that circadian regulation affects photosynthesis, but photosynthesis also affects the core structure of the circadian clock [10].

Circadian regulation affects stomatal behaviour and the proportion of the diurnal oscillation in stomatal conductance (*g*_s_) that is currently attributed to the clock, amounting to 30–35% of the total daytime variation [4,11], is larger than the previously clock-attributed variation in photosynthesis. Stomatal conductance, thus, appears to be under a stronger circadian regulation than photosynthesis, but the function of circadian regulation of stomatal behaviour has not yet been adequately assessed (but see [12]).

Stomatal opening is necessary for the diffusion of CO_2_ from the atmosphere into the mesophyll, but this comes at a water cost. A long-term standing evolutionary model on stomatal function proposes that stomata operate optimally to balance the ratio between C assimilation (*A*) and transpiration (*E*), that is, to maximise water use efficiency (WUE). In short, the hypothesis of optimal stomatal behaviour proposes that stomata maintain a constant marginal water cost (*λ* = δ*E*/δ*A*; in mol CO_2_ mol^−1^ H_2_O), at least over short time scales, at the point where *A* balances the cost of water lost through *E* [13].

Recent studies propose alternative optimization strategies. For instance, Wolf et al. [14] hypothesized that leaves maximize *A* minus hydraulic risks that are a function of tissue water potential (*A−*Θ(ψ)), such as xylem impairment, which would incur significant fitness or carbon costs. The traditional WUE hypothesis proposes that plants follow a conservative strategy to save water, whereas Wolf et al. [14] propose that plants prioritize carbon maximization over water savings (CM hypothesis), which is consistent with plant competition for water in the soil.

Circadian biologists have addressed how the clock regulates water use efficiency [15] and they often mention circadian regulation as important for attaining optimal stomatal conductance [16]. However, we are unaware of any direct tests for stomatal optimality incorporating circadian regulation. Circadian regulation of *A* has been documented to be uncoupled and independent from circadian regulation of *g*_s_ [17,18], but linkages between these two processes are a prerequisite for optimal WUE. Therefore, if circadian rhythms regulate *A* and *g*_s_ independently from each other, this suggests that circadian regulation alone would not lead to optimal stomatal regulation or, at least, not directly.

Nonetheless, there is some evidence from theoretical modelling that circadian rhythms could aid in reaching optimality. Circadian regulation serves to “anticipate” predictable environmental cues, in such a way that stomata can adjust prior to experiencing the environmental condition (“stomatal priming”, [5]). As such, the clock has been hypothesized to aid in attaining optimality through stomatal priming because direct responses to regular diurnal fluctuations alone would inevitably lead to a lagged response [19]. In other words, since stomata show a lagged response to the environment [20] and although it is not expected that optimality operates at every instant, circadian regulation could help in achieving optimality by diminishing the lags [19].

In describing the WUE hypothesis, Cowan [21] states that “if diurnal variation in natural physical environment were regular and predictable, then optimization would require only that there be an appropriate circadian rhythm in stomatal aperture”. Given that variation in the physical environment is not entirely regular and predictable, here, we seek to understand whether the function of circadian regulation in stomatal behaviour contributes to optimal stomatal behaviour. More specifically, we wanted to test whether circadian regulation would lead to optimal stomatal behaviour, as predicted by the WUE hypothesis or, alternatively, whether stomatal optimization via circadian regulation would be more consistent with the CM hypothesis. A secondary objective was to understand the implications of our findings for stomatal modelling. More specifically, we sought to understand the effects of circadian regulation on the slope of a commonly used Ball–Berry type of stomatal model [22].

Assessing the effects of circadian regulation on daytime *A* and *g*_s_ under natural conditions is difficult because the influence of environmental drivers generally mask circadian regulation. Circadian regulation is most strongly expressed under a free-running “constant environment”: when temperature, radiation, vapour pressure deficit, and other environmental drivers are held experimentally constant over 24 h or longer. Therefore, we addressed our questions by examining temporal variation in gas exchange and stomatal behaviour in an herb (bean, *Phaseolus vulgaris*) and in a shrub (cotton, *Gossypium hirsutum*) under 48 h of constant environmental conditions.

## 2. Materials and Methods

### 2.1. Experimental Set-Up

The experiment was performed at the Macrocosms platform of the Montpellier European Ecotron, Centre National de la Recherche Scientifique (CNRS, France). We used 6 controlled-environment units of the macrocosms platform (three planted with bean and three with cotton), where the main abiotic (air temperature, humidity, and CO_2_ concentration) drivers were automatically controlled. Intact soil was extracted using large cylindrical lysimeters (2 m^2^, circular with a diameter of 1.6 m and a depth of 2 m, weighing 7 to 8 tonnes) from the flood plain of the Saale River near Jena, Germany. The lysimeters were brought to Montpellier Ecotron and used in a previous experiment on grassland biodiversity [23]. Following that experiment, the soil was ploughed down to 40 cm following standard agricultural practice and fertilized with 25/25/35 NPK (MgO, SO_3_, and other oligoelements were associated in this fertilizer: Engrais bleu universel, BINOR, Fleury-les-Aubrais, FR).

The soil was regularly watered to ca. field capacity by drip irrigation, although irrigation was stopped during each measurement campaign (few days) to avoid interference with water flux measurements. No differences in leaf water potential were observed (*p* ≤ 0.05; paired *t*-test, *n* = 3) between the beginning and end of these measurement campaigns, indicating no apparent effect of a potentially declining soil moisture on leaf hydration.

Environmental conditions within the macrocosms (excluding the experimental periods) were set to mimic outdoor conditions but did include a minor (10%) light reduction by the macrocosm dome cover (sheet of Fluorinated Ethylene Propylene). During experimental periods, light was controlled by placing a completely opaque fitted cover on each dome to block external light inputs (PVC coated polyester sheet Ferrari 502, assembled by IASO, Lleida, Spain), and by using a set of 5 dimmable plasma lamps (GAN 300 LEP with the Luxim STA 41.02 bulb, with a sun-like light spectrum); these lamps were hung 30 cm above the plant canopy and provided a photosynthetically active radiation (PAR) at a canopy level of 500 μmol m^−2^ s^−1^(Li-190, LI-COR Biosciences, Lincoln, NE, USA). PAR was chosen to be 500 μmol m^−2^ s^−1^ because previous research proposed that stomatal behaviour should follow optimal theory when photosynthesis is light- (and not CO_2_) limited [22].

Monocultures of bean and cotton were planted in each macrocosm, along 5 rows with 30 cm distance between the rows, on 10 July 2013, one month before the start of the measurements, and were thinned to densities of 10.5 and 9 individuals m^−2^, respectively (about 30 cm interplant distance on the row). Cotton (STAM-A16 variety by the Institut National des Recherches Agricoles du Bénin/Centre de coopération internationale en recherche agronomique pour le développement, INRAB/CIRAD) is a perennial woody shrub with an indeterminate growth habit. This cotton variety grows to 1.5–2 m tall and has a pyramidal shape and short branches. Bean (recombinant inbred line RIL-115 bred by INRA (Institut National de la Recherche Agronomique) Eco & Sol) is an annual herbaceous species. RIL-115 is a fast growing, indeterminate dwarf variety, 0.3–0.5 m tall; it was inoculated with Rhizobium tropici CIAT 899, also provided by INRA. During the experiment, bean and cotton generally remained at the inflorescence emergence developmental growth stage codes 51–59 in BBCH scale, the standard phenological scale within the crop industry [24,25]. More detailed information on Ecotron measurements can be found elsewhere [26].

During each experimental period, plants were entrained for five days under environmental conditions that mimicked the pattern observed in an average August sunny day in Montpellier in terms of air temperature (*T*_air_, 28/19 °C, diurnal max/min) and vapor pressure deficit (VPD), and at PAR of 500 μmol m^−2^ s^−1^, as discussed above. After 5 days of entrainment, we maintained constant environmental conditions starting at solar noon and for the next 48 h.

### 2.2. Measurements

Gas exchange measurements were conducted during 48 h of constant environmental conditions. We measured CO_2_ and water vapor exchanges every 2 min by using 2–3 portable photosynthesis systems (LI-6400XT, Li-cor Inc, Lincoln, USA) per species and day [21]. Plants were one month old, about 30 cm tall, and we selected fully expanded leaves from the upper portion of the canopy. Each instrument was continuously deployed on a leaf for 24 h, and the Auto-Log function was used. Measurements were conducted over 48 h with an effective *n* = 3 per species (1–2 leaves were measured per macrocosm, in a total of 3 macrocosms).

### 2.3. Analyses

Data collected during the 48 h free-running period were pooled together into a single 24 h period for analyses to increase statistical power. We modelled temporal patterns in gas exchange using Generalized Additive Models (GAM). GAMs provided a flexible tool that is very well suited to analyse temporal patterns because it does not include predefined functional structures. To test for statistical significance in the temporal pattern, we computed the first derivative of the best-fit trend line following [27].

We tested for optimization of *g*_s_ under circadian regulation following the approach developed by Anderegg et al. [28]. That is, we fit the *g*_s_ data for each species to find the set of parameters (*λ*; for WUE and Θ′ for CM, where Θ′ = a ψ_L_ + b and ‘a’ and ‘b’ are fitted parameters) that best explains the observed variation in *g*_s_. In the model in Anderegg et al. [28], hydraulic transport is simulated via the supply–demand approach in Sperry and Love [29] with a single whole-plant resistor characterized by the stem hydraulic vulnerability curve; photosynthesis is simulated via the standard Farquhar et al. [30] photosynthesis model; and the two are linked via either the WUE or CM optimization equations. This model takes in the environmental drivers of the hydraulic (pre-dawn water potential) and photosynthesis (atmospheric CO_2_, PAR, leaf temperature, and VPD) and predicts *g*_s_. Following Anderegg et al. [28], a Markov Chain Monte Carlo approach is used for finding the values of *λ* or Θ′ that best predict the observed *g*_s_ values.

Finally, we addressed the modelling implications of our findings by using our data to calibrate the stomatal model from Medlyn et al. [22]. This model is based on the WUE hypothesis and, importantly, it includes one parameter (*g*_1_) that is directly related to the marginal water use efficiency. Therefore, consistent with the WUE hypothesis, we expected *g*_1_ to remain constant during our experiment.

## 3. Results

**Figure 1:**
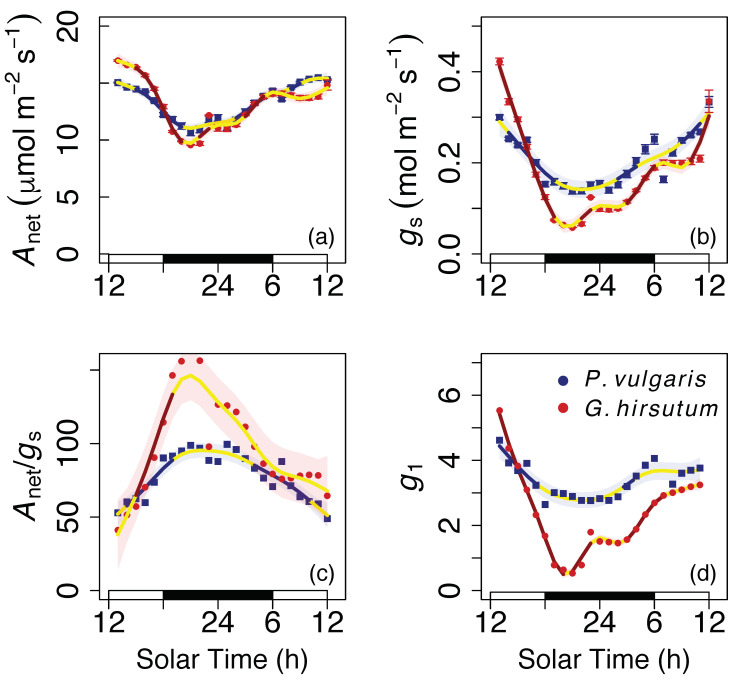
Circadian oscillation in gas exchange. The dots (with small SE bars hidden) indicate hourly averages of (**a**) assimilation (*A*_net_), (**b**) stomatal conductance (*g*_s_), (**c**) the ratio between the two (*A*_net_/*g*_s_), and (**d**) a parameter proportional to the marginal water cost of carbon gain (*g*_1_). Measurements were conducted under constant environmental conditions (see Figure 2). The white and black rectangles at the base indicate the subjective day (when it would have been daytime during entrainment) and subjective night, respectively, under constant conditions. Lines (and shaded error intervals) indicate the prediction (and SE) of Generalized Additive Model (GAM) fitting separately for each species (some lines may overlap), and portions which are not yellow indicate significant temporal variation.

**Figure 2:**
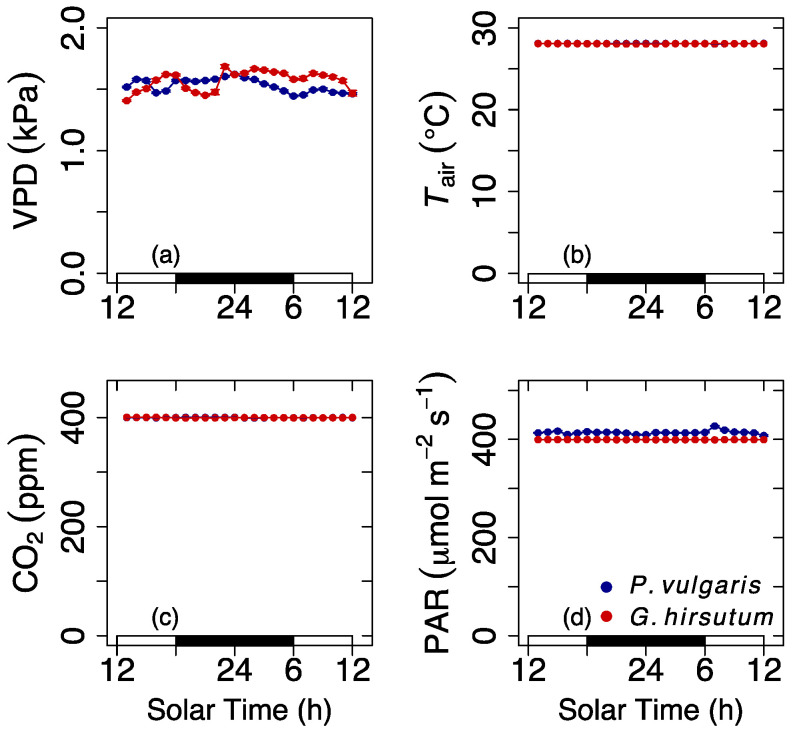
Environmental conditions during gas exchange measurements. The dots (with small SE bars hidden) indicate hourly averages of (**a**) vapor pressure deficit (*VPD*), (**b**) air temperature (*T*_air_), (**c**) CO_2_ concentration, and (**d**) photosynthetically active radiation (*PAR*). Conditions in the leaf cuvette mirrored those in the macrocosms. The white and black rectangles at the base indicate the subjective day (when it would have been daytime during entrainment) and subjective night, respectively, under constant conditions. Some values may be hidden.

We observed a self-sustained oscillation in *A*_net_, *g*_s_, and *A*_net_/*g*_s_ that showed a ~24 h period (Figure 1 and Figure 2). That is, there was a significant variation in *A*_net_ and *g*_s_ in the absence of variation in environmental drivers during the free-running period, and this variation showed a ~24 h periodicity. *A*_net_ varied from 10.7 (at 21.00 h, solar time) to 15.5 μmol m^−2^ s^−1^ (at 11.00 h) in bean and from 9.6 (at 21.00 h) to 17.0 μmol m^−2^ s^−1^ (at 13.00 h) in cotton. *g*_s_ varied from 0.14 (at 22.00 h) to 0.33 mol m^−2^ s^−1^ (at 11.00 h) in bean and from 0.06 (at 22.00 h) to 0.42 mol m^−2^ s^−1^ (at 13.00 h) in cotton. Furthermore, if we only consider the oscillation during the subjective day (the time under constant conditions when it would have normally been daytime during entrainment), we still observe a significant and time-dependent variation in *A*_net_, *g*_s_, and *A*_net_/*g*_s_, although of smaller magnitude than during the whole 24 h cycle (Figure 1 and Figure 2).

The pattern in *A*_net_/*g*_s_ was such that water use efficiency increased in the first subjective afternoon (hours 12–18 in Figure 1c) under constant conditions from 52.9 to 90.2 in bean and from 41.0 to 90.4 in cotton. *A*_net_/*g*_s_ remained constant (between 91 and 88) during the first hours of the night in bean, but it continued to increase (until 164.8) in cotton. *A*_net_/*g*_s_ decreased in both species from the subjective midnight until the following subjective noon (Figure 1c).

**Figure 3:**
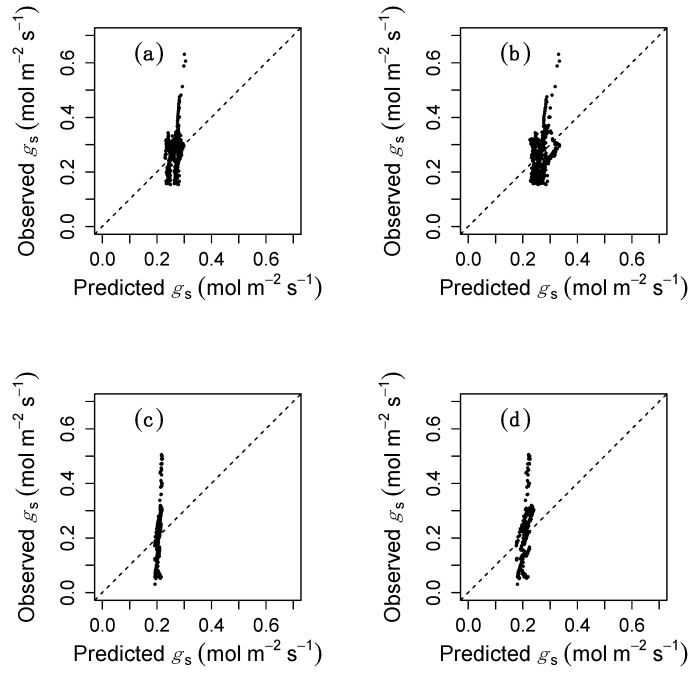
Results of model fitting. Observed vs. predicted values of *g*_s_ for bean (**a**,**b**) and cotton (**c**,**d**) under the hypotheses that stomata operate to optimize water use efficiency (**a**,**c**) or to maximize carbon assimilation (**b**,**d**).

We observed that *g*_s_ was poorly predicted by the WUE and the CM hypotheses (Figure 3). For the case of bean, *g*_s_ oscillated between 0.15 and 0.63 mol m^−2^ s^−1^. However, predictions from the WUE hypothesis varied between 0.23 and 0.30 mol m^−2^ s^−1^ and predictions from the CM hypothesis varied between 0.22 and 0.33 mol m^−2^ s^−1^. Similarly, *g*_s_ in cotton oscillated between 0.03 and 0.51 mol m^−2^ s^−1^, whereas predicted *g*_s_ ranged between 0.19 and 0.22 mol m^−2^ s^−1^ for the WUE hypothesis and between 0.17 and 0.23 mol m^−2^ s^−1^ for the CM hypothesis.

Finally, we observed a significant temporal variation of the parameter *g*_1_ from the Medlyn et al. [22] model: between 2.64 and 4.61 in bean and between 0.53 and 5.53 in cotton (Figure 1c).

## 4. Discussion

We observed a significant and self-sustained 24 h oscillation in *A*_net_ and *g*_s_, of different magnitude for each process, and that ultimately led to a diurnal oscillation in intrinsic water use efficiency (*A*_net_/*g*_s_). Moreover, we observed that the oscillation of *g*_s_ could not be predicted by current optimization models, suggesting that, contrary to conventional wisdom, circadian regulation does not directly lead to optimal stomatal behaviour or, alternately, that circadian regulation may provide benefits (i.e., be evolutionarily optimal) but current optimization models may not account for a key mechanism or cost to capture this behaviour.

There are many processes that could explain an afternoon decline in *A*_net_, including feedback inhibition from starch accumulation, photorespiration as well as stomatal feedbacks, amongst others [31,32,33]. Similarly, a multitude of processes could explain the afternoon decline in *g*_s_, including hydraulic feedbacks and depletion of stem capacitors [32,34]. However, the only process that can explain a self-sustained 24 h cycle is the circadian clock [35].

Current optimization schemes failed to capture the observed variation in *g*_s_ because they assume a major role for environmental conditions. Consequently, in the absence of significant environmental variation, the models predicted nearly constant *g*_s_, which is in sharp contrast with our results. This does not imply that the WUE or the CM hypotheses are necessarily wrong: those hypotheses were developed to explain *g*_s_ in a normally varying environment. However, we interpret poor model fit as an indication that circadian regulation does not directly lead to optimal stomatal behaviour, at least not as defined by the current optimization schemes.

We observed a stronger relative fluctuation in *g*_s_ than in *A*_net_, consistent with previous studies [4,11]. These temporal patterns could be interpreted as an indication that the clock fosters a maximization of *A* at the time of maximal potential for assimilation (*A* peaked at the subjective noon) which, in turn, would be aided by a maximal *g*_s_ which decreases diffusional limitations. On the other hand, the stronger decrease in *g*_s_, relative to that in *A*_net_, during the subjective morning and afternoon, when conditions would have become less favourable for assimilation in a naturally fluctuating environment, is consistent with a conservative water use strategy. Therefore, this result is consistent with the hypothesis that circadian-driven stomatal priming could contribute towards reaching optimality [19], although through indirect effects. Further studies would need to address the potential for such indirect effects and to aggregate circadian effects at daily timescales.

Circadian regulation of stomatal conductance may have some implications for modelling. The stomatal model proposed by Medlyn et al. [22] would predict a unique and temporally constant value of *g*_1_ for a given species. Using this approach, Lin et al. [36] examined global variation in *g*_1_ across 314 species and observed significant differences when grouping plants into different plant functional types. That is, *g*_1_ varied from 1.6 to 7.2 for different plant functional types and subsequently, Kala et al. [37] proposed using different values of *g*_1_ for different plant functional types to improve land surface modelling. Within our 24 h dataset, *g*_1_ varied from 0.5 to 5.5 (between 2.3 and 5.5 during the subjective day). That is, we observed in one species and for one day, a variation in *g*_1_ that is of similar magnitude to that observed in a global synthesis. This result indicates that measurements to calibrate stomatal models may need to take time of day into consideration to account for potential artifacts from circadian regulation.

It is worth noting that the environmental conditions that plants experienced in this experiment were not completely unrealistic for a field setting. While having light at night is obviously implausible, it is not uncommon for plants in some environments to experience cloudy afternoons where PAR remains around 500 μmol m^−2^ s^−1^ and where *T*_air_ and VPD do not show much environmental variation [38]. Our observation that the highest variation in *g*_1_ occurred around that time is intriguing, as it suggests some variation within field settings could occur as well. We, thus, encourage further field studies of leaf level gas exchange at high temporal resolution to further understand a possible temporal variation in *g*_1_.

More broadly, our results indicate that the evolutionary significance underlying circadian regulation of stomata is still unknown. We have demonstrated that circadian *g*_s_ is not consistent with predictions from the WUE or CM models, which offer different perspectives on the evolution of stomatal behaviour. Hence, we need an alternative evolutionary framework that explains our observations under circadian action. Considering that circadian regulation leads to a large diurnal oscillation in *g*_s_ (30–35%), we expect that response to be adaptive. We argue that future progress will be made by integrating circadian stomatal regulation over daily and even seasonal scales. That is, we need to jointly analyse nocturnal and diurnal circadian stomatal conductance and to additionally consider links between stomata and photoperiod responses [39,40]. Overall, we also need to more explicitly incorporate endogenous circadian rhythms into our understanding of *g*_s_ variation in a naturally varying environment.
